# Lifestyle factors and obesity in young adults – changes in the 2000s in Finland

**DOI:** 10.1177/14034948221075427

**Published:** 2022-02-08

**Authors:** Tuija Jääskeläinen, Päivikki Koponen, Annamari Lundqvist, Seppo Koskinen

**Affiliations:** Finnish Institute for Health and Welfare, Department of Public Health and Welfare, Helsinki, Finland

**Keywords:** Young adults, lifestyle, overweight, obesity, population-based study

## Abstract

**Aims:**

Young adulthood is a life stage that is vulnerable to detrimental lifestyle changes and excessive weight gain, which may have major effects on health later in life. This study aimed to examine the changes in lifestyle-related factors in the 2000s and sociodemographic differences in lifestyle in Finnish young adults.

**Methods:**

The study was based on the cross-sectional data from two representative samples of Finnish young adults aged 18−29 years from the Health 2000 Survey (*n* = 1894; 90% participated) and the FinHealth 2017 Study (*n* = 1162; 54% participated). Sociodemographic factors, lifestyle choices (smoking, alcohol consumption, intake of vegetables, physical activity), and anthropometrics were self-reported. Weighted prevalence based on predictive margins and odds ratios were analyzed using logistic regression, taking into account the sampling design and non-response.

**Results:**

The prevalence of daily cigarette smoking decreased between the years 2000 and 2017 from 34% to 12% (*p* < 0.01) and from 23% to 11% (*p* < 0.01) in men and women, respectively. There was a decline in the prevalence of daily intake of fresh vegetables, especially in men. The prevalence of obesity (BMI ⩾ 30 kg/m^2^) doubled being 15% in men and 18% in women in 2017. Health-endangering lifestyles, measured by a lifestyle sum score, were more common among young adults with lower education compared to those with higher.

**Conclusions:**

**This study showed both favorable and unfavorable changes in the lifestyles of Finnish young adults in the 2000s. Health-endangering lifestyles were more common among young adults with lower education, suggesting the need for tailored health-promoting actions. Special attention should be given to obesity prevention.**

## Background

Young adulthood, covering approximately the ages from 20 to 30, is a pivotal transition period from adolescence to adulthood typically involving several significant life changes related to education, working life, and social relationships [1]. Establishing independence may also have an impact on health-related lifestyles. A health-endangering lifestyle, smoking, abundant alcohol consumption, a poor diet, physical inactivity, and obesity, is a major risk factor for several burdensome chronic conditions like diabetes [2] and cardiovascular diseases [3], Epidemiological evidence in adults has shown that lifestyle-related factors are associated with health outcomes both individually and when combining lifestyle factors to give an overall lifestyle score; in general, the larger the number of health-endangering lifestyle factors, the higher the risk of adverse health outcomes [2-4].

Chronic diseases are more prevalent among middle-aged or older adults than young adults, but adolescence and young adulthood are critical periods in which to adopt healthy lifestyles and to lay the foundation for future health [1,5]. Young adults are prone to making detrimental lifestyle changes like a decline in physical activity levels [6]. Furthermore, young adulthood is a vulnerable period in relation to excessive weight gain and the development of obesity [5]. These negative changes in lifestyle-related factors may have major effects on health later in life. For example, results based on over 100,000 US men and women from the Health Professional follow-up study and the Nurses’ Health study showed that weight gain from early to middle adulthood was associated with an increased risk of major chronic disease and a decreased likelihood of healthy aging [7].

Recently published reports in the UK [8,9] and the US [1] have pointed to major lifestyle-related health concerns, for example, increasing obesity rates in young adults. However, encouraging changes, like falling trends in the prevalence of daily cigarette smoking, have also been observed. Therefore, up-to-date information on the lifestyles of young adults is needed to develop more effective health-promotion strategies. Several previous studies have focused on specific target groups, like university students [10,11]. Information based on population-based samples is scarce. Further, it has been indicated that health-endangering lifestyle factors typically co-occur in the same individual [12] and accumulate, especially in young adults with lower education [13]. Thus, it is important to examine multiple lifestyle factors and their accumulation, taking into account the sociodemographic characteristics.

## Aims

The aim of this study was to provide population-based information on five lifestyle-related factors: smoking, alcohol consumption, diet, physical activity, and obesity, and to highlight changes over the last two decades in Finnish young adults. Further, this study aimed to examine whether there are sociodemographic differences in lifestyle-related factors as well as in the co-occurrence of multiple health-endangering lifestyle-related factors among young adults.

## Methods

This study is based on cross-sectional data from two Finnish population-based surveys coordinated by the Finnish Institute for Health and Welfare, which were conducted in 2000–2001 (the Health 2000 Survey; H2000) [14] and in 2017 (the FinHealth 2017 Study; FH17) [15]. In both years, a two-stage stratified cluster sample of young adults aged 18–29 years was drawn from the nationwide population register in Finland. The sampling design of FH17 was based on that of H2000 in order to obtain nationally representative data in both years. The H2000 sample of young adults consisted of 1894 individuals. A total of 90% (*n* = 1710) of them participated in the health interview and/or returned a questionnaire. The FH17 sample included a total of 1162 young adults of whom 54% (*n* = 625) returned a questionnaire or participated in the short telephone interview.

H2000 was approved by the Ethical Committee for Research in Epidemiology and Public Health in May 2000, and FH17 was approved by the Coordinating Ethics Committee in March 2016 at the Hospital District of Helsinki and Uusimaa. Informed consent was obtained from all participants.

In both studies information on age and sex was obtained from the Population Register Centre of Finland. Health interviews (H2000), self-administered questionnaires (H2000 and FH17), and a short telephone interview (FH17) provided information on other sociodemographic- and lifestyle factors as well as collating data on weight and height. In H2000, the number of missing values for single variables in this study varied from 4 to 460 due to differences in data collection modes: interview, full self-administered questionnaire, or only a short questionnaire. In FH17, the number of missing values for single variables varied between 4 and 38.

### Sociodemographic factors

Age was dichotomized: 18–24 and 25–29 years old. The participants were asked about their current main activity (employee or self-employed, unemployed, student, retired, on family leave, or other). The highest completed education degree was elicited and for the analyses the participants were dichotomized: 1) basic or secondary vocational education or 2) general upper-secondary education, bachelor’s degree or higher.

### Lifestyle-related factors

The participants were asked about their smoking status (cigarettes, cigars, pipes), which was dichotomized into daily cigarette smokers vs. others. Daily use of smokeless tobacco (snus) was assessed by a similar question. Those who described their alcohol consumption with the answer option “I have been a non-drinker all my life (or tasted alcohol not more than 10 times during my life)” were defined as non-drinkers. Further, the frequency of alcohol consumption was dichotomized into those who consumed alcohol at least once a week vs. others (including non-drinkers). Those consuming fresh vegetables or root vegetables (excluding potatoes, fruit, and berries) at least six to seven times per week were defined as daily vegetable users. The question concerning leisure-time physical activity included four categories:1 – physical inactivity, 2 – moderate everyday exercise several hours a week, 3 – vigorous exercise several hours a week, 4 – competitive sport, and for the analysis these were dichotomized into physically inactive during leisure time (Category 1) vs. active (Categories 2-4). Commuting physical activity was assessed by asking how many minutes participants travel on foot, by bicycle, or similar on their way to work or school. The participants were dichotomized into two classes: no commuting physical activity (including those not working/studying) or commuting physical activity less than 15 min daily vs. others. Weight and height were self-reported. BMI was calculated as weight divided by height squared. Obesity was classified as BMI ⩾ 30 kg/m^2^ and overweight as BMI ⩾ 25 kg/m^2^, as defined by World Health Organization for adults (aged ⩾ 20 years) [16] because the majority of participants were at least 20 years old.

The lifestyle score was modified from the criteria presented by Khera et al., for example [3]. Due to the limitation that a comparable variable indicating high-risk use of alcohol was not available for both study years, in the present study health-endangering lifestyle was defined based on four key lifestyle-related factors as follows: 1) daily cigarette smoking, 2) intake of fresh vegetables less frequently than daily, 3) physically inactive during leisure time, 4) BMI ⩾ 30 kg/m^2^ (obesity). For each component, participants who met the criterion for a health-endangering lifestyle received 1 point, while those who did not meet the criterion were scored 0. Thus, the total score ranged from 0 to 4, with higher scores suggesting an unhealthier lifestyle.

**Figure 1.**
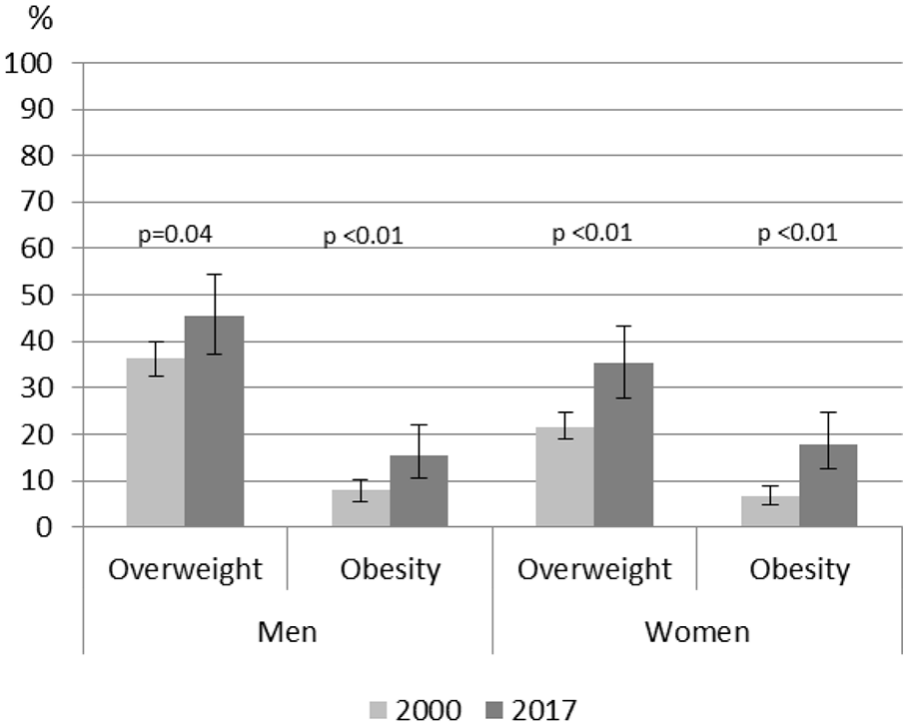
Weighted prevalence (%) and 95% confidence intervals of overweight and obesity in 2000 and 2017 stratified by sex. Values are based on predictive margins analyzed using a logistic regression model. The *p*-values refer to the difference between the years 2000 and 2017 (Wald’s test). Overweight BMI ⩾ 25kg/m^2^; obesity BMI ⩾ 30kg/m^2^.

All statistical analyses were carried out using SAS 9.3 [17] and SUDAAN 11.0.1 [18] taking into account the sampling design. Inverse probability weights [19] were used in all analyses (excluding characteristics of the study populations presented in Table I) to adjust for differences in selection probability, to reduce the bias due to non-participation, and to provide nationally representative results. Weighted prevalence based on predictive margins [20] and odds ratios was analyzed using logistic regression (binary response variables) or multinomial logistic regression (nominal response variables). Tests of statistical significance for differences between the study years (Table II, Figure 1) were carried out using Wald’s test. The analyses were either stratified by sex (Table II, Figure 1) or adjusted for sex (Table III). When analyzing the association between the lifestyle factors and educational degree (Table III) participants aged < 20 years were excluded because, in general, they have not finished their secondary education.

**Table I.** Characteristics of the study populations: unweighted values based on descriptive statistics.

	Health 2000	FinHealth 2017
Study years	2000	2017
Sample, *n*	1894	1162
Participation^a^, *n* (%)	1710 (90)	625 (54)
Men, *n* (%)	862 (50)	295 (47)
Age, mean (SD)	23 (4)	26 (3)
Educational degree^b^, *n* (%)		
Basic education	97 (7)	27 (5)
Vocational secondary education	430 (31)	161 (28)
General upper-secondary education	453 (33)	128 (22)
Bachelor’s degree or higher	401 (29)	261 (45)
Main activity, *n* (%)		
Employee or self-employed	1006 (59)	337 (55)
Student	367 (21)	157 (25)
Unemployed or retired	165 (10)	73 (12)
Other	170 (10)	49 (8)

a Health 2000: Health interview and/or full questionnaire or short questionnaire; FinHealth 2017 full questionnaire or short telephone interview.

b Participants aged <20 were excluded.

**Table II.** Weighted prevalence (%) and 95% confidence intervals (CI) of the lifestyle factors in 2000 and 2017 based on predictive margins analyzed using logistic or multinomial logistic regression model.

	Men					Women				
	Health 2000		FinHealth 2017			Health 2000		FinHealth 2017		
	%	95% CI	%	95% CI	*p* ^a^	%	95% CI	%	95% CI	*p* ^a^
Smoking										
Daily smoking of cigarettes	33.8	30.3, 37.5	11.5	7.1, 18.2	<0.01	22.6	19.5, 26.1	11.0	7.8, 15.2	<0.01
Daily use of snus	3.4	2.3, 4.9	8.3	4.8, 14.1	0.01	NA		NA		NA
Daily smoking of cigarettes and/or snus	36.7	33.1, 40.4	18.6	13.1, 25.7	<0.01	22.6	19.5, 26.1	12.0	8.8, 16.3	<0.01
Alcohol consumption										
Non-drinkers	10.8	8.3, 13.9	18.3	12.8, 25.4	0.01	13.2	10.8, 16.0	17.2	11.5, 25.0	0.23
At least weekly a drink containing alcohol	13.1	10.6, 16.1	9.1	6.0, 13.4	0.10	6.6	4.9, 9.0	4.7	2.6, 8.3	0.31
Diet										
Fresh vegetables less frequently than daily	54.6	51.0, 58.3	67.8	59.3, 75.2	0.01	42.2	38.4, 46.2	47.8	40.5, 55.2	0.20
Physical activity										
Physically inactive during leisure time	26.7	23.2, 30.5	21.3	15.1, 29.1	0.18	26.3	23.3, 29.6	25.8	19.7, 33.0	0.88
Commuting physical activity: not at all or <15 min/day	65.1	61.1, 68.9	64.4	55.1, 72.8	0.89	51.5	47.6, 55.3	57.9	49.4, 65.9	0.17
Lifestyle score^b^										
0 Health-endangering lifestyle factor	26.3	22.3, 30.7	28.4	22.2, 35.6		38.6	34.8, 42.5	36.1	29.7, 43.1	
1 Health-endangering lifestyle factor	41.1	36.3, 46.1	41.3	36.5, 46.3		35.0	30.8, 39.4	35.3	31.2, 39.7	
2 Health-endangering lifestyle factors	23.5	20.0, 27.5	22.0	17.7, 27.1		18.9	15.9, 22.2	20.2	16.1, 24.9	
3−4 Health-endangering lifestyle factors	9.1	6.7, 12.2	8.2	5.6, 11.9	0.51	7.6	5.8, 10.0	8.4	5.5, 12.5	0.53

NA: not available due to low number of snus users.

a For the difference between the years 2000 and 2017, Wald’s test.

b Health-endangering lifestyle factors: daily smoking of cigarettes, fresh vegetables less frequently than daily, physically inactive during leisure time, body mass index ⩾ 30 kg/m^2^ (obesity).

**Table III.** Weighted odds ratios (ORs) and their 95% confidence intervals (CIs) for the lifestyle-related factors according to sociodemographic characteristics analyzed using logistic regression model.

	Daily smoking of cigarettes		Fresh vegetables less frequently than daily		Physically inactive during leisure time		Body mass index ⩾ 30 kg/m^2^ (obesity)		⩾ 2 Health-endangering lifestyle factors^a^	
	OR	95% CI	OR	95% CI	OR	95% CI	OR	95% CI	OR	95% CI
Health 2000										
Men vs. women (ref.)	1.75	1.37, 2.22	1.65	1.31, 2.07	1.02	0.80, 1.30	1.23	0.81, 1.87	1.57	1.23, 2.00
Aged 18−24 vs. 25−29 years (ref.)^b^	1.12	0.89, 1.40	1.23	0.98, 1.53	1.01	0.78, 1.32	0.68	0.47, 1.01	1.15	0.90, 1.48
Basic or secondary vocational education vs. higher education (ref.)^b,c,d^	3.46	2.62, 4.56	1.94	1.51, 2.48	2.41	1.79, 3.24	1.87	1.20, 2.90	3.64	2.75, 4.82
FinHealth 2017										
Men vs. women (ref.)	1.05	0.56, 1.98	2.30	1.45, 3.65	0.78	0.46, 1.32	0.85	0.47, 1.53	0.91	0.55, 1.52
Aged 18−24 vs. 25−29 years (ref.)^b^	0.93	0.51, 1.67	1.38	0.89, 2.15	1.17	0.71, 1.91	0.89	0.51, 1.57	1.10	0.65, 1.85
Basic or secondary vocational education vs. higher education (ref.)^b,c,d^	4.00	2.00, 7.98	2.84	1.75, 4.61	1.61	0.90, 2.89	2.25	1.40, 3.62	2.35	1.44, 3.85

a Health-endangering lifestyle factors: daily smoking of cigarettes, fresh vegetables less frequently than daily, physically inactive during leisure time, body mass index ⩾ 30 kg/m^2^ (obesity).

b Adjusted for sex.

c Participants aged <20 were excluded.

d Higher education: general upper-secondary education, bachelor’s degree, or higher.

## Results

In both years, about half of the participants were men (Table I). In 2017, participants were on average older compared with 2000: the mean age in 2000 was 23 whereas in 2017 it was 26 years. Over 60% of those aged 20 or over had general upper-secondary education, bachelor’s degree, or higher in both studies. More than half of the participants were employees or self-employed in both study years.

The prevalence of daily cigarette smoking decreased remarkably between the years 2000 and 2017: from 34% to 12% and from 23% to 11% in men and women, respectively (Table II). In men, the prevalence of daily use of snus increased from 3% to 8%. The prevalence of non-drinkers increased slightly in both sexes between the study years, but the change was statistically significant only for men. There was a decline in the prevalence of daily intake of fresh vegetables, especially in men. In 2017, 68% of men and 48% of women consumed fresh vegetables less frequently than daily. There were no significant changes in leisure-time or commuting physical activity between the study years. In both years, about one in four young adults was physically inactive during their leisure time and over 60% of young men and over 50% of women commuted under 15 min daily by foot or bicycle.

Overweight and obesity increased in both sexes between the years 2000 and 2017 (Figure 1). In 2017, 46% of young men and 35% of young women were overweight (BMI ⩾ 25 kg/m^2^). The prevalence of obesity doubled being 15% and 18% in men and women, respectively, in 2017.

In both years, about a quarter of young men and a third of young women did not have any of the following health-endangering lifestyle factors: current daily cigarette smoking, intake of fresh vegetables less frequently than daily, being physically inactive during their leisure time, or being obese (BMI < 30 kg/m^2^) (Table II). There were no changes in the distribution of the number of health-endangering lifestyle factors between the study years. Intake of fresh vegetables less frequently than daily was the most common health-endangering lifestyle factor in both years. Concerning the combinations of two health-endangering lifestyle factors, intake of vegetables less frequently than daily and physical inactivity during leisure time most commonly co-occurred, about 10% of young adults having this combination in both years (2000: 9% (95% CI 7,12) of men, 9% (7,11) of women; 2017 10% (6,18) of men, 9% (5,15) of women). Among the combinations of three health-endangering lifestyle factors, intake of fresh vegetables less frequently than daily combined with physical inactivity and current smoking was the most common combination in 2000 (7% (95% CI 5,9) of men; 4% (95% CI 3,6) of women). In 2017, current smoking was replaced with obesity, reflecting the changes in the prevalences of smoking and obesity between the study years.

In 2000, men were more likely to be daily cigarette smokers (OR 1.75; 95% CI 1.37, 2.22) compared to women (Table III). In 2017, there was no significant difference in the prevalence of daily cigarette smoking between the sexes (OR 1.05; 95% CI 0.56, 1.98). Further, in both study years men were less likely to consume fresh vegetables daily. Lower education (i.e. basic or secondary vocational education) was associated with greater odds of being a daily cigarette smoker, consuming fresh vegetables less frequently than daily, and being obese as well, as having multiple health-endangering lifestyle factors in both study years.

## Discussion

### Major findings

The present study based on two cross-sectional Finnish nationally representative samples showed that there have been both favorable and unfavorable changes in the health-related lifestyles of young adults in the 2000s. The prevalence of daily cigarette smoking has decreased, but the rising prevalence of overweight and obese young adults is alarming. Further, based on the lifestyle sum score, there were no changes in the distribution of the number of health-endangering lifestyle factors in the same individual between the study years. Health-endangering lifestyle factors were found to accumulate especially in young adults with low education in both study years.

In line with previous findings on UK young adults [9] we observed a decline in the prevalence of daily cigarette smoking among Finnish young adults during the 2000s. In Finland, this is probably mainly due to the effective tobacco policy over the last decades [21]. At the same time, however, the daily use of snus has become more common among Finnish young men, indicating that preventive actions to reduce the use of tobacco products are still needed. Further, we found an increase in the prevalence of non-drinkers; however, this was statistically significant only for men. The same phenomenon has been observed in England where the prevalence of non-drinking among young adults aged 16–24 years increased from 18% to 29% between 2005 and 2015 [22].

Our results confirm previous findings [9] that the rising prevalence of overweight and obese young adults is a current major and increasing public health concern. Compared to other Nordic countries, our results are in line with the Norwegian Students’ Health and Wellbeing Study, which showed that the prevalence of overweight among university students aged 26−34 years in 2018 was 49% and 37% in men and women, respectively [10]. About 14% of the Norwegian university students were obese. Further, the prevalence of overweight among Swedish young adults aged 18–34 increased from 33% to 42% and the prevalence of obesity from 7% to 13% between the years 1995 and 2017 [23]. These findings point to the urgent need to focus on obesity prevention in young adulthood, especially since this period has been shown to be vulnerable to rapid weight gain [5]. It is noteworthy that, among children and adolescents (5–19 years), the increasing trend in BMI has plateaued in many high-income countries since 2000s [24].

In the present study, we observed a decline in the daily intake of fresh vegetables especially in young men, which may indicate negative changes in the dietary habits and be associated with the rising obesity rates [25]. Regarding physical activity, we did not find any significant changes in the levels of leisure-time or commuting physical activity between the study years. It is possible, however, that sedentary behavior has increased [26], which lowers the levels of energy expenditure and may also be associated with negative changes in dietary habits in young adulthood [27].

Health-endangering lifestyle factors typically cluster [12]. In the present study, over a quarter of the young adults had at least two out of four health-endangering lifestyle-related factors in 2017 but we did not observe indications that the accumulation of health-endangering lifestyle factors in the same individuals had increased between the study years. Further, in line with previous findings [13], we observed that thealth-endaring lifestyle factors accumulated in those young adults with lower education. Previous research has also shown that young men tend to have unhealthier lifestyles compared with women [13]. We did not find any gender differences in the co-occurrence of multiple health-endangering lifestyle factors in 2017, but daily intake of fresh vegetables was significantly lower among young men than women.

Finally, our study highlighted the importance of monitoring and examining health and its amenable determinants regularly in young adulthood at the population level. Due to lower chronic disease incidence among young adults compared to older adults too little attention is typically paid to them. Recently, the need for population-based health examination surveys has also been identified in American young adults [28].

### Methodological issues

The major strength of this study was that it was based on two nationally representative cross-sectional samples with similar sample designs, allowing the examination of changes in the lifestyles of young adults over the last two decades at the population level. Further, there was the possibility to examine a wide range of lifestyle factors that were determined with the same validated methods in both years.

As to limitations, the study sample was smaller and the participation rate was lower in FH17 compared to H2000 [14,15]. Further, there was a difference in the age distribution of the study samples: the FH17 sample included relatively fewer young adults aged 18−24 compared to the H2000 sample. Thus, the participants were on average older in 2017 than in the 2000 study. We used inverse probability weights [19] to adjust for differences in selection probability, to correct the effects of non-participation, and to improve the generalizability of the results to the Finnish population, but it is nevertheless possible that the lower participation rate in FH17 may have caused some bias to the results. The participation rate in FH17 was, however, higher than several other studies carried out in this age group in recent years [10].

Regarding lifestyle variables, it was not possible to compare high-risk use of alcohol between the years 2000 and 2017 due to major differences in the questions concerning alcohol consumption between the study years. Thus, only the prevalence of non-drinkers and those using alcohol at least once a week were reported. Finally, in this age group, measured weight and height were available in FH17 only because the H2000 study protocol for young adults did not include a health examination. Thus, we used self-reported information on weight and height in both studies to ensure comparability of the methods between the study years. It is well-known that self-reported height is typically overestimated and weight underestimated [29]. It has been shown, however, that self-reported anthropometric information on young adults is a valid basis for classifying participants according to BMI [30].

## Conclusions

In conclusion, this Finnish nationally representative study showed both favorable and unfavorable changes in the lifestyles of young adults over the last two decades. The prevalence of daily cigarette smoking has decreased but there has been an increase in the use of snus among young men. The prevalence of overweight and obesity has increased considerably. Health-endangering lifestyles were more common among young adults with lower education. The results suggest that there is a need for young-adult-focused health-promotion efforts that are tailored to different population groups. Special attention should be given to obesity prevention in young adulthood.
